# RNF4 Regulates the BLM Helicase in Recovery From Replication Fork Collapse

**DOI:** 10.3389/fgene.2021.753535

**Published:** 2021-11-12

**Authors:** Nathan Ellis, Jianmei Zhu, Mary K Yagle, Wei-Chih Yang, Jing Huang, Alexander Kwako, Michael M. Seidman, Michael J. Matunis

**Affiliations:** ^1^ University of Arizona Cancer Center, University of Arizona, Tucson, AZ, United States; ^2^ Department of Biochemistry and Molecular Biology, Bloomberg School of Public Health, Johns Hopkins University, Baltimore, MD, United States; ^3^ Laboratory of Molecular Gerontology, National Institute on Aging, Baltimore, MD, United States

**Keywords:** Bloom syndrome, BLM, DNA repair, dormant origins, fork collapse, homologous recombination, hydroxyurea, RAD51

## Abstract

Sumoylation is an important enhancer of responses to DNA replication stress and the SUMO-targeted ubiquitin E3 ligase RNF4 regulates these responses by ubiquitylation of sumoylated DNA damage response factors. The specific targets and functional consequences of RNF4 regulation in response to replication stress, however, have not been fully characterized. Here we demonstrated that RNF4 is required for the restart of DNA replication following prolonged hydroxyurea (HU)-induced replication stress. Contrary to its role in repair of *γ*-irradiation-induced DNA double-strand breaks (DSBs), our analysis revealed that RNF4 does not significantly impact recognition or repair of replication stress-associated DSBs. Rather, using DNA fiber assays, we found that the firing of new DNA replication origins, which is required for replication restart following prolonged stress, was inhibited in cells depleted of RNF4. We also provided evidence that RNF4 recognizes and ubiquitylates sumoylated Bloom syndrome DNA helicase BLM and thereby promotes its proteosome-mediated turnover at damaged DNA replication forks. Consistent with it being a functionally important RNF4 substrate, co-depletion of BLM rescued defects in the firing of new replication origins observed in cells depleted of RNF4 alone. We concluded that RNF4 acts to remove sumoylated BLM from collapsed DNA replication forks, which is required to facilitate normal resumption of DNA synthesis after prolonged replication fork stalling and collapse.

## Introduction

Accurate DNA replication is essential to maintenance of genome integrity. When the replicative polymerase encounters DNA damage such as chemical modifications of bases, the polymerase stalls at the site of the DNA lesion and the CDC45-MCM2-7-GINS (CMG) helicase uncouples from the polymerase and continues to unwind downstream duplex to expose single-stranded DNA (ssDNA) (Cortez, 2019). ssDNA binding protein (RPA) binds to ssDNA, and the complex activates the ATR kinase, which is required for the recruitment of factors from the homologous recombination (HR) pathway (Dungrawala et al., 2015). These factors stabilize and protect the replication fork from nascent-strand degradation (Schlacher et al., 2011). Fork protection and stabilization are dependent on the RAD51 recombinase (Zellweger et al., 2015; Mijic et al., 2017), which is thought to engage with other factors, including BRCA2 and SMARCAL1, that reverse the fork to form a structure resembling a Holliday junction and prevent nascent strand degradation due to exonucleolytic attack (Hashimoto et al., 2010; Kolinjivadi et al., 2017).

Nucleotide deprivation using the ribonucleotide reductase inhibitor hydroxyurea (HU) has provided a powerful probe to examine the mechanisms of fork stabilization and protection because no specific DNA lesion is generated (Vesela et al., 2017). Uncoupling occurs due to DNA polymerases stalling on the template following a >50% decrease in purine deoxynucleotide concentration (Skoog and Bjursell, 1974). In this context, activation of ATR and the recruitment of HR factors, such as RAD51 and BRCA2, are replication-specific (Petermann et al., 2010; Zellweger et al., 2015). Early studies conducted with radiolabeled thymidine tracer demonstrated that ribonucleotide reductase inhibition achieved a cessation of DNA replication and, upon removal of HU, DNA replication resumed (Bianchi et al., 1986). Analysis of DNA replication dynamics based on single molecule DNA fiber assay has demonstrated that HU-stalled replication forks resume synthesis at the sites where they stalled (Petermann et al., 2010; Sidorova et al., 2013; Thangavel et al., 2015). However, prolonged treatment with HU (≥16 h) leads to irreversible fork collapse. Cells treated with HU ≥16 h have two cell populations, namely, cells that were in S phase when drug was added to the medium and cells that transited the cell cycle then entered and were stalled at the beginning of S phase (Karnani and Dutta, 2011). Because collapsed forks cannot be restarted, successful genome duplication in cells depends on the firing of dormant origins (Woodward et al., 2006; Ge and Blow, 2010). The majority of dormant origins fired in these cells are >100 kb away from the collapsed forks.

Multiple proteins that are associated with replication fork stability and HR are sumoylated (Xiao et al., 2015). Sumoylation is in turn regulated by recruitment of SUMO-targeted ubiquitin E3 ligases (STUbLs). STUbLs contain tandem SUMO interaction motifs (SIMs), which bind poly-sumoylated proteins, and RING domains that mediate ubiquitylation (Prudden et al., 2007). The prototypical mammalian STUbL, RNF4, was first identified as a transcriptional co-regulator of hormone receptors (Moilanen et al., 1998). The *S. cerevisiae* ortholog of mammalian RNF4, the Slx5/Slx8 heterodimer, was discovered in a screen for genes synthetically lethal with the *BLM* ortholog *Sgs1* (Mullen et al., 2001). Yeast strains lacking *Slx5* or *Slx8* are hypersensitive to chronic DNA replication stress and exhibit elevated levels of gross chromosome rearrangements and spontaneous mutations (Mullen et al., 2001; Zhang et al., 2006; Prudden et al., 2007); *Slx5* or *Slx8* regulate the levels of numerous sumoylated HR proteins (Burgess et al., 2007). In mammalian cells, RNF4 has been found to operate in a variety of DNA repair functions. It controls the formation of double-strand breaks (DSB) in ATR-deficient cells undergoing replication stress (Ragland et al., 2013). It mediates the recruitment of BRCA1 to DSB sites through the generation of SUMO-ubiquitin hybrid chains (Guzzo et al., 2012). It is recruited to sites of DNA damage *via* sumoylated MDC1 and is required for exonucleolytic processing of DSBs preceding HR-mediated repair (Galanty et al., 2012; Luo et al., 2012; Yin et al., 2012). RNF4-mediated ubiquitylation facilitates the extraction of proteins from DNA repair sites through recruitment of the Cdc48/p97 segregase (Nie et al., 2012), it regulates FANCI/FANCD2 turnover at stalled forks (Gibbs-Seymour et al., 2015), and it mediates the release of FAAP20 from sumoylated FANCA during interstrand crosslink repair (Xie et al., 2015). Although the function of RNF4 in DSB repair has been studied by many laboratories, it’s role in responding to fork collapse has not been well characterized in mammalian cells.

The BLM helicase has been implicated in replication fork stability as *BLM-*deficient cells exhibit multiple defects in DNA replication, including accumulation of abnormal DNA replication intermediates (Lonn et al., 1990), slower replication fork velocity (Rao et al., 2007), and excessive firing of dormant origins (Davies et al., 2007). *BLM-*deficient cells exhibit increased levels of chromatid breakage and HR, in particular, elevated sister chromatid exchanges (SCEs) (Chaganti et al., 1974). BLM interacts directly with both RAD51 (Wu et al., 2001; Bugreev et al., 2007; Patel et al., 2017) and RPA (Brosh et al., 2000; Doherty et al., 2005; Xu et al., 2008; Shorrocks et al., 2021). We have shown previously that BLM’s function in DNA replication is regulated by sumoylation (Ouyang et al., 2009). Cells that express a sumoylation-deficient BLM (SD-BLM) accumulate lower levels of RAD51 at collapsed forks but higher levels of RPA (Eladad et al., 2005; Ouyang et al., 2009; Ouyang et al., 2013). Study of an RPA-binding-deficient BLM showed that BLM’s capacity to bind RPA is required for its role in fork protection (Shorrocks et al., 2021), but the mechanism is unclear because RPA can block BLM’s DNA unwinding activity *in vitro* (Xue et al., 2019). Levels of SCE are normal in both untreated SD-BLM cells (Eladad et al., 2005) and untreated RPA-binding-deficient BLM cells (Shorrocks et al., 2021); however, in SD-BLM cells subject to prolonged HU treatment, SCEs are not induced but instead DSBs accumulate, associating the failure to recruit RAD51 to collapsed forks with failure to repair DSBs that are generated there. Our recent work with mutations of *NSMCE2*, the SUMO E3 ligase responsible for BLM sumoylation at collapsed forks, suggests that at least some of these DSBs occur subsequent to dormant origin firing when converging forks merge with collapsed forks (Pond et al., 2019).

Because BLM’s functions are regulated by sumoylation, we hypothesized that RNF4 promotes turnover of BLM at collapsed replication forks, possibly to facilitate downstream DSB processing events. We show here that sumoylated BLM is indeed an RNF4 substrate, but RNF4 is not required for DSB repair at collapsed forks. Instead, we found that deficiency in BLM turnover at collapsed forks resulted in defects in dormant origin firing.

## Materials and Methods

### Cell Culture and Transfection

For knockdown experiments, cells were cultured to 30–50% confluence and transfected with Lipofectamine RNAiMAX (Invitrogen). NC1 negative control siRNA was obtained from IDT, Inc., RNF4 siRNA #1 (5’-GAC​TCA​CAA​TGA​CTC​TGT​TGT​GAT​T-3’) from Invitrogen, and RNF4 siRNA #2 (5’-GAA​UGG​ACG​UCU​CAU​CGU​UUU-3’) from Dharmacon. SENP6 siRNA (5’-AAG​AAA​GTG​AAG​GAG​ATA​CAG​UU-3’) was obtained from Qiagen, Inc. and BLM siRNA (5’- UCC​CGG​GAU​ACU​GCU​CUC​A-3’) was from IDT, Inc. siRNAs were used at a final concentration of 20 nM.

### Antibodies

Antibodies were obtained from the following sources: anti-RPA/p34 (Neomarkers MS-691-P0), anti-*γ*-H2AX (Millipore 05-636), rabbit anti-BLM (Eladad et al., 2005), rabbit anti-RNF4 (a gift from Dr. Jorma Palvimo), rabbit anti-CHK1 (Cell Signaling Technology 2345) at 1:400, rabbit anti-phospho-CHK1 (ser317) (Cell Signaling Technology 2344S) at 1:400, rat anti-HSC70 (Assay Design) at 1:45,000, anti-tubulin (Sigma T9026), anti-SUMO2 (Zhang et al., 2008), anti-Myc (Cell Signaling Technology 2276S), and anti-SENP6 (a gift from Dr. Mary Dasso). AlexaFluor-labeled secondary antibodies (A11029; A11035), were obtained from Invitrogen. HRP-labeled secondary antibodies were anti-mouse IgG (Cell Signaling Technology 7076S), anti-rat IgG (Jackson Labs), and anti-rabbit IgG (GE Healthcare NA934V). For DNA fiber assays, antibodies for detection of 5-iodo-2′-deoxyuridine (IdU) were mouse anti-IdU (BD) and for detection of 5-chloro-2′-deoxyuridine (CldU) were rat anti-CldU (Abcam); the secondary antibodies were anti-mouse Dylight 488 and anti-rat Dylight 649 (Jackson ImmunoResearch).

### Clonogenic Survival Assay

U2OS or HeLa cells were transfected with control or RNF4 siRNAs. 48 h after transfection, cells were incubated with varying concentrations of HU for 72 h or of camptothecin (CPT) for 3 h. Cells were trypsinized after treatment and counted with a hemocytometer. Then, 200, 400, and 800 cells were seeded in duplicate into six-well or 60 mm plates in normal medium. After one to two weeks, clones with >50 cells were scored. Clonogenic survival was calculated as the average of number of clones over the number of cells seeded for all scorable wells or dishes. The results were normalized to the clonogenic survival of untreated, negative control condition. The experiment was repeated three times. The average of experiments and standard deviations were calculated.

### Immunofluorescence Microscopy

For analysis of RNF4 localization, U2OS cells were seeded in 35-mm glass-bottom culture dishes. For HU-induced DNA damage, cells were incubated in the presence of 2 mM HU for 16 h. Laser-induced irradiation was performed as described (Muniandy et al., 2009). Irradiated cells were allowed to recover for 2 h before antibody staining. Cells were fixed and stained as described (Zhang et al., 2008). Images were collected using Zeiss Observer Z1 fluorescence microscope with an Apotome VH optical sectioning grid and processed using AxioVision Software Release 4.8.1.

For analysis of DNA repair foci numbers and focal areas, 50,000 HeLa cells were seeded on cover slips, transfected with siRNAs, and treated with 2 mM HU for 16 h. As a positive control for a known RNF4-dependent phenotype, we also treated cells with 10 μM etoposide for 4 h, which is known to generate DSBs in all cell cycle phases. Following treatment, cells were pre-processed as described (Dimitrova and Gilbert, 2000) then fixed and stained as described (Zhang et al., 2008). Images were collected on a Zeiss LSM 710 Meta Confocal microscope using Zeiss LSM4.2 software. Foci numbers and areas were acquired in ImageJ/FIJI. The DAPI image was used to generate the nuclear regions of interest. For each experiment an image threshold of the red and green channels was determined using the brightest conditions (e.g., HU-treated control), and this threshold was used for all images acquired. Particles >2 pixels were counted in the resulting binary images using the Analyze Particles function. Quantification of numbers of foci was carried out using CellProfiler (version 2.0). 30 to 70 cells were counted in each experiment. Three experiments were performed and the three experiments were combined and box and whiskers plots prepared from the merged data.

### Pulsed-Field Gel Electrophoresis to Measure Double-Strand Breaks

One million HeLa or U2OS cells were seeded into 35 mm dishes. The next day cells were transfected with negative control or RNF4 siRNAs. 48 h after transfection of siRNAs, the cells were treated with varying concentrations of CPT for 3 h. The cells were harvested, counted, and 250,000–3,000,000 cells were embedded in agarose plugs in agarose insert buffer (10 mM Tris-HCl pH 7.5, 20 mM NaCl, 50 mM EDTA). Plug preparation, cell lysis, and agarose gel electrophoresis were carried as described (Ouyang et al., 2009; Pond and Ellis, 2019). Gels were stained with SYBR Gold (1 part in 10,000 parts water) and the UV transilluminator image was analyzed using ImageJ Gel Analyzer. Arbitrary fluorescence units were normalized to untreated, untransfected controls. Experiments were repeated three times. The average of experiments and standard deviations were calculated.

### Flow Cytometric Analysis

300,000 U2OS or HeLa cells or 150,000 HCT116 cells were seeded into 40 mm dishes and forward transfected the following day with negative control or RNF4-specific siRNAs. 24–30 h after transfection, the cells were treated or not with 2 mM HU for 16 h. The cells were released from the HU block and incubated in media containing 20 μM BrdU for 20, 30, 40, 60, or 120 min prior to harvest. 20 min of BrdU labeling was used for each time point prior to harvest. Processing of cells for flow cytometry was carried out using the APC BrdU Flow kit (BD Pharmingen) according to the manufacturer’s instructions. The fixed and stained cells were analyzed on a Beckman Coulter Cyan ADP. Data was analyzed using Summit 4.3 software from Beckman Coulter. After gating, percent of cells in each cell-cycle phase (G1, S, and G2/M) was calculated. Experiments were repeated three or more times. The average of experiments and standard deviations were calculated.

### DNA Fiber Analysis

U2OS cells were transfected with control or RNF4 siRNAs. 48 h after transfection, cells were exposed to 20 μM IdU for 20 min. Cells were incubated or not with 2 mM HU for 2 or 16 h. Cells were washed and then exposed to 100 μM CldU for 30 min. DNA fibers were prepared and visualized as described (Davies et al., 2007). Microscopy was carried out using a Zeiss Observer Z1 fluorescence microscope.

### Analysis of BLM Sumoylation and RNF4-Mediated Ubiquitylation

U2OS cells stably transfected with His-SUMO-1 or His-SUMO-2 were a gift from Dr. Mary Dasso (NIH). His-SUMO conjugates were purified as described (Jaffray and Hay, 2006). For *in vitro* sumoylation and ubiquitylation reactions, recombinant GST-tagged BLM (amino acids 1-431), SUMO E1 (Aos1/Uba2), E2 (Ubc9), and SUMO proteins were expressed and purified from *E. coli* as previously described (Zhu et al., 2008). Recombinant ubiquitin E1 (Uba1), E2 (UbcH5c), RNF4, and ubiquitin were kindly provided by Dr. Cynthia Wolberger (Johns Hopkins University). Sumoylated BLM on GST beads was produced as previously described (Zhu et al., 2008). The GST-BLM-SUMO beads were washed and incubated with 1 μM ubiquitin E1, 25 μM UbcH5a and 1 mM ubiquitin with or without 50 μM RNF4 in reaction buffer [1 mM ATP, 20 U/ml creatine phosphokinase, 5 mM phosphocreatine, 0.6 mg/ml inorganic pyrophosphatase in 20 mM HEPES-KOH (pH 7.3), 110 mM potassium acetate, 2 mM magnesium acetate and 1 mM DTT] at 37**°**C for 2 h. After five washes with 500 mM NaCl in PBS, proteins were eluted with 2X SDS sample buffer and analyzed by immunoblot analysis.

### Analysis of BLM Stability

Cells were transfected with negative control or RNF4 siRNA oligos on two sequential days. 24 h after the second transfection, cells were treated with or without 100 ng/ml cycloheximide for different times and lysed directly in sample buffer at the end of treatment. Proteins were analyzed by immunoblot analysis.

### Mitotic Index Analysis

Mitotic index was measure by seeding 50,000 transfected HeLa or HCT116 cells onto cover slips and treating them the next day with 2 mM HU for 16 h. Cells were released from the HU block by replacement with fresh medium, and the cover slips were processed for examination of mitoses at 1-h time points 9–15 h after release. For processing, cells were fixed in 4% paraformaldehyde diluted into PBS at room temperature for 20 min, washed in PBS, permeabilized in 0.5% Triton X-100 for 10 min, washed 3 times in PBS (the middle wash containing 0.1 M glycine), and mounted in Prolong Gold with DAPI (Invitrogen). Cells were examined at 40x magnification with a UV filter on a Nikon Eclipse E600 controlled by NIS-Elements BR 3.0 software. Fields were imaged and mitotic cells were identified as cells with chromosomes undergoing condensation or cells containing condensed chromosomes. The percent of mitotic cells was calculated as the total number of mitotic cells divided by the total number of cells. A small percentage of cells (<0.2%) that exhibited nuclear blebbing or other nuclear changes indicative of apoptosis, were not included in this total. Experiments were repeated three times. The average of experiments and standard deviations were calculated z.

### Sister Chromatid Exchange

165,000 HeLa cells were seeded into T25 flasks overnight and were subsequently transfected with siRNAs on two sequential days. On the third day, cells were incubated in medium containing 20 μM bromodeoxyuridine (BrdU) for 42 h (untreated samples) or cells were incubated in 20 μM BrdU for 24 h, in 20 μM BrdU and 2 mM HU for 16 h, and finally in 20 μM BrdU for 14 h (HU-treated samples). 45 min prior to harvest, colcemid was added to achieve a concentration of 0.15 μg/ml. For HU-treated cultures, 3 h prior to harvest freshly prepared caffeine was added at a concentration of 1 mM, otherwise mitoses were not obtained in RNF4-depleted cells. The failure to obtain mitoses in RNF4-depleted cells was specific to colcemid treatment, because RNF4-depleted cells underwent mitosis and cell division in the absence of colcemid. Metaphases were prepared and stained using the fluorescence plus Geimsa method as described (Ouyang et al., 2009). Metaphases were examined at 100x magnification under oil with a Nikon Eclipse E600 controlled by NIS-Elements BR 3.0 software. SCEs in each metaphase and the numbers of chromosomes were counted, and the levels of SCEs expressed as the number of exchanges per 46 chromosomes over all scorable metaphases. Experiments were repeated twice. The average of experiments and standard deviations were calculated.

### Statistical Analyses

T-tests were performed to compare the effects of RNF4 depletion vs control on proliferation; on focal accumulations of RAD51, *γ*-H2AX, BLM, and RPA; on SCEs; and, on replication dynamics exhibited by the DNA fiber analysis. To compare flow cytometry cell cycle profiles, the difference in the mean values for RNF4 versus NC1 were tested for percentage phase of the cell cycle (% G1, % S, and % G2) within each cell line using two sample *t*-tests. The interaction between caffeine and RNF4 (versus NC1) was initially tested using linear regression. Since it was not statistically significant for any conditions, it was removed from the model. Subsequently the difference between the caffeine treatment in the no HU and HU conditions was assessed using two sample *t*-tests. To analyze recovery from replication arrest, the difference in the percent of cells between siRNF4 and control was assessed using linear regression models. The initial model assessed whether the profile of the change across time differed between the two conditions (i.e., was there interaction). If the profile was not significantly different, the potential for a shift between the two conditions was assessed.

## Results

### RNF4 Exhibited a Unique Role in Response to Prolonged Replication Stress

**FIGURE 1:**
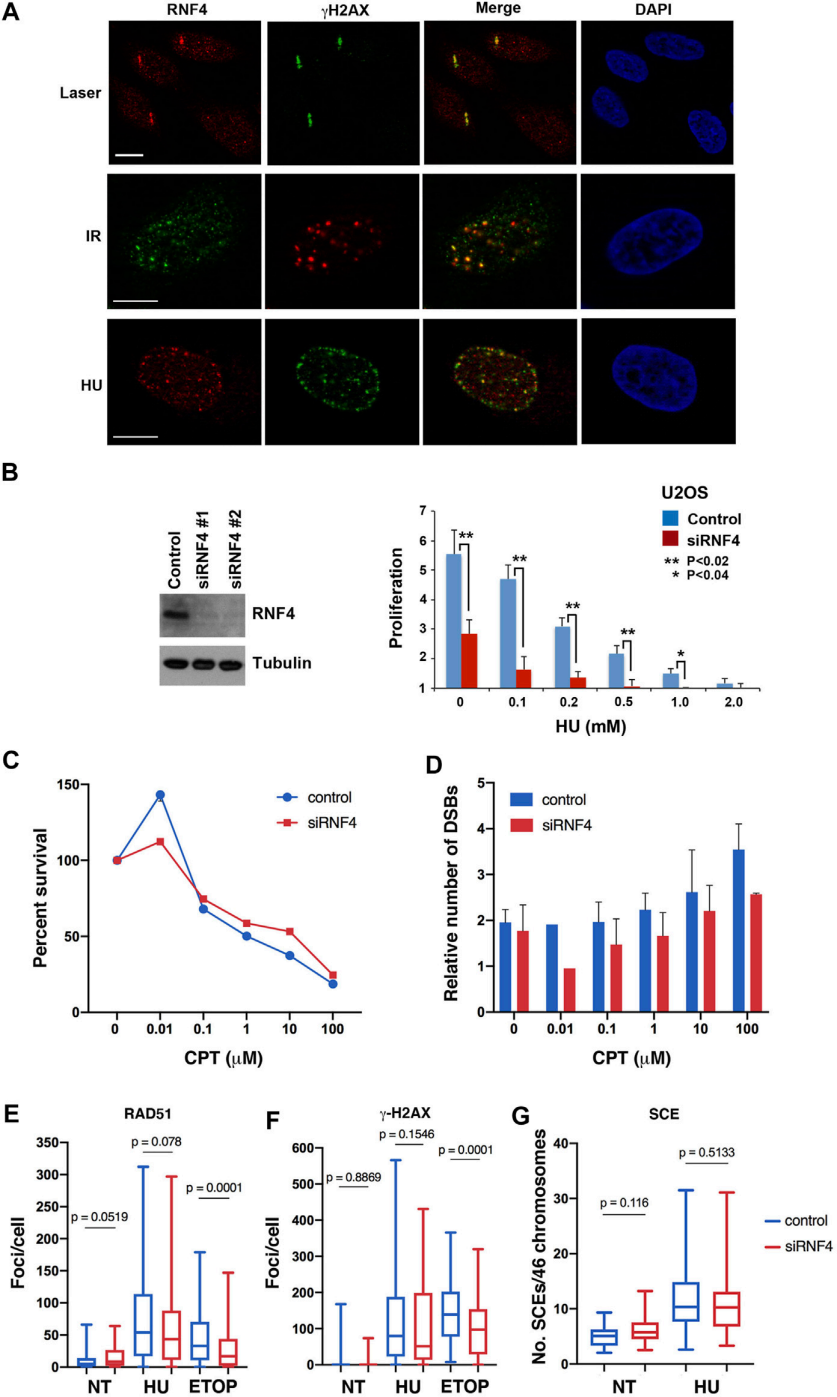
RNF4 was not required for HR repair of replication-associated DSBs. **(A)** RNF4 co-localized with phosphorylated histone H2AX (*γ*-H2AX) in U2OS cells treated with a micro-laser, ionizing radiation (IR), or hydroxyurea (HU). DNA was detected with DAPI. Bars = 10 µm. **(B)** RNF4 depletion in U2OS cells by two unique RNF4-specific siRNAs was evaluated by immunoblot analysis. Proliferation defect in RNF4-depleted U2OS cells exposed to varying concentrations of HU for 72 h. Error bars represent standard deviations from three biological replicates. *p* values were determined using a Student’s *t*-test. **(C)** Line graph showing results of clonogenic survival assays on RNF4-depleted and control-depleted HeLa cells exposed to varying concentrations of camptothecin (CPT) for 3 h. Results from three independent assays were averaged and standard deviations shown. **(D)** Bar graphs showing the relative levels of DSBs in RNF4-depleted and control-depleted HeLa cells exposed to varying concentrations of CPT for 3 h, as determined by pulsed field gel electrophoresis. Induced DSBs were normalized to DSBs in untransfected and untreated cells. Results from three independent experiments were averaged and standard deviations shown. **(E,F)** Box and whiskers plots showing the enumerations of focal accumulations of RAD51 and *γ*-H2AX, detected by indirect immunofluorescence, in RNF4-depleted and control-depleted HeLa cells untreated or treated 2 mM HU for 16 h. Results from three independent experiments were combined. As a positive control, cells were treated with 10 μM etoposide for 4 h. **(G)** Box and whiskers plot showing levels of sister chromatid exchange (SCE) in RNF4-depleted and control-depleted HeLa cells untreated or treated 2 mM HU. Cells were labeled with BrdU for one cell division, blocked in HU for 16 h, released from the HU block into medium containing BrdU, collected in metaphase with colcemid and processed for assaying SCEs. Results from two independent experiments were combined. NT = not treated.

Although known to have important roles in cellular response to DNA replication stress (Ragland et al., 2013; Kumar and Sabapathy, 2019), the exact functions of RN4 at stalled and collapsed replication forks are not fully understood. To explore these functions, we first used indirect immunofluorescence confocal microscopy to investigate RNF4 localization in U2OS cells treated with 2 mM HU for 16 h, as localization under these conditions has not previously been reported. This analysis revealed that RNF4 co-localized with *γ*-H2AX, a marker for sites of collapsed replication forks and the modicum of DSBs induced by this duration of HU treatment (Petermann et al., 2010). As positive controls, we confirmed previous findings that RNF4 co-localizes with *γ*-H2AX at DSBs generated by microlaser- and *γ*-irradiation (Galanty et al., 2012; Yin et al., 2012; Vyas et al., 2013) (Figure 1A).

To further explore the functions of RNF4 in the cellular response to DNA replication stress, we used two independent siRNA oligos to effectively deplete expression levels by >90% in human U2OS cells (Figure 1B). Consistent with previous studies (Galanty et al., 2012; Yin et al., 2012; Vyas et al., 2013), we found that RNF4 depletion enhanced sensitivity to chronic HU-induced replication stress (Figure 1B; Supplementary Figure S1A). We also examined the sensitivity of RNF4-depleted HeLa and U2OS cells to CPT, a topoisomerase I (TopI) inhibitor that stabilizes the normally transient TopI cleavage complex. CPT treatment also induces DNA replication stress in S phase, and the collision of replication forks with CPT-TopI-DNA complexes induces formation of DSBs (Vesela et al., 2017). We found that RNF4-depletion had no effect on the sensitivity to CPT (Figure 1C; Supplementary Figure S1B). Because CPT toxicity is linked to generation of DSBs, we measured the number of CPT-induced DSBs in HeLa and U2OS cells by pulsed-field gel electrophoresis. This analysis revealed that DSB formation was also unaffected in RNF4-depleted cells compared to control cells (Figure 1D; Supplementary Figure S1C). These observations suggested that RNF4 plays a unique role in the response to DNA replication stress, and that this role involves functions at sites of collapsed replication forks that may be independent of DSB repair.

RNF4 functions in the repair of DSBs generated by *γ*-irradiation in part by facilitating the recruitment of RAD51 to sites of DNA damage (Galanty et al., 2012; Yin et al., 2012; Vyas et al., 2013). We therefore examined whether RNF4 depletion in HeLa cells impaired RAD51 accumulation at collapsed replication forks and DSBs generated by prolonged HU treatment. Unexpectedly, the numbers of RAD51 and *γ*-H2AX foci, as well as their focal areas, were similar in RNF4-depleted cells with or without HU treatment compared to control-depleted cells (Figures 1E,F; Supplementary Figure S1D), suggesting normal responses to collapsed replication forks. In contrast, and as expected, the number of RAD51 foci were reduced in RNF4-depleted HeLa cells treated with etoposide, an inhibitor of Topoisomerase II that induces DSBs directly in all phases of the cell cycle (Vesela et al., 2017). Contrary to expectation, *γ*-H2AX foci were also reduced, suggesting that RNF4 depletion leads to slower proteolytic processing of the TopII-DNA cleavage complex or of the tyrosyl-DNA moiety left over after proteolysis (Sciascia et al, 2020). The tyrosyl-DNA moiety is processed by tyrosyl-DNA phosphodiesterase 2, which is a target of sumoylation and regulated by RNF4 (Sun et al., 2020). We also measured rates of SCE in HU-treated HeLa cells, which serves as a readout for DSB repair through RAD51-dependent HR. In agreement with the normal RAD51 recruitment to DSBs, the levels of SCE were similar in RNF4-depleted compared to control-depleted cells with or without HU treatment (Figure 1G). These findings provided further evidence that RNF4 plays a unique role in the response to HU-induced replication stress that is distinct from DSB recognition and repair.

### RNF4 was Required for Normal Recovery of DNA Synthesis After Prolonged Replication Stress

**FIGURE 2:**
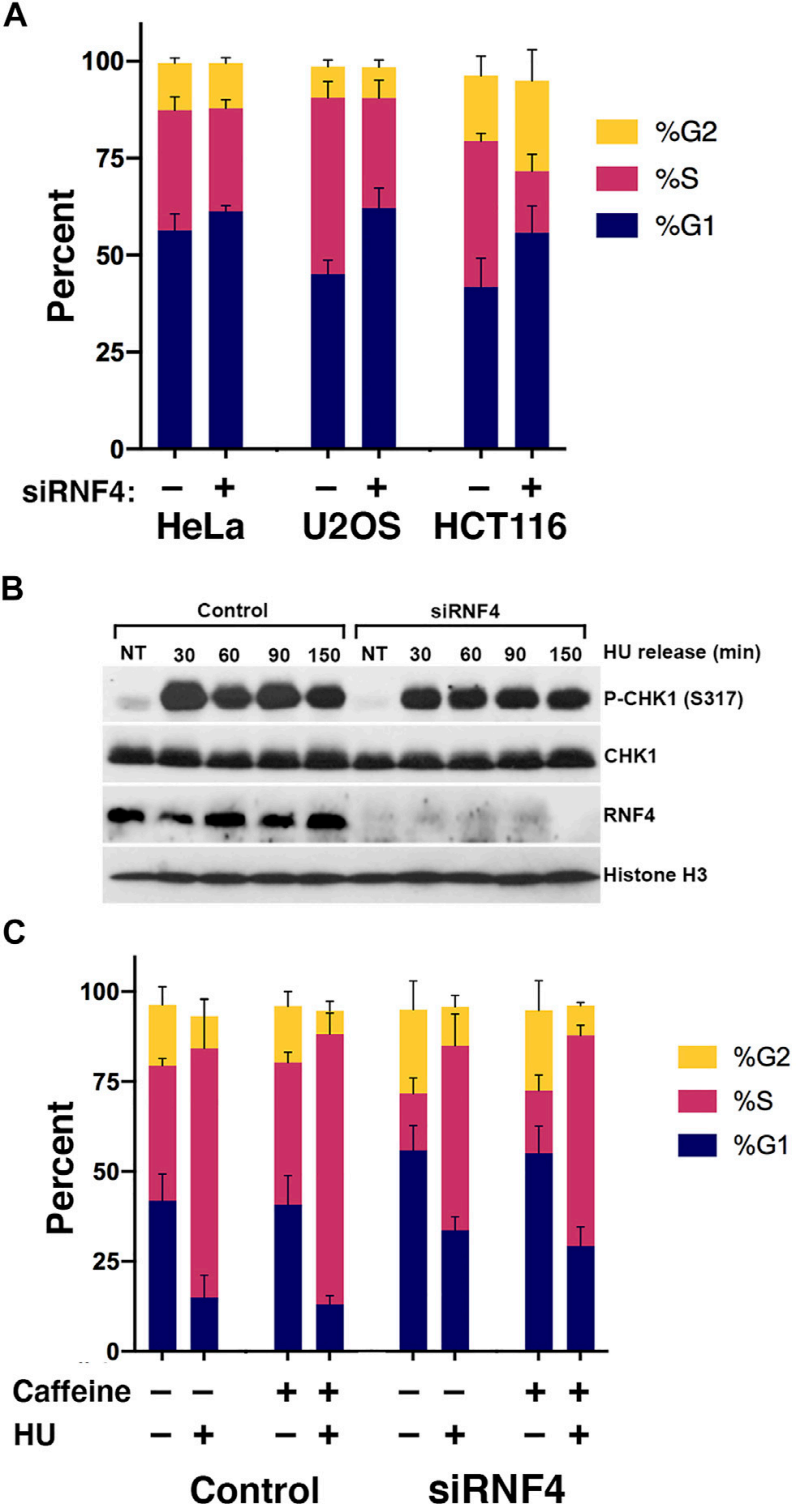
RNF4 depletion was associated with a cell cycle defect. **(A)** RNF4-depleted cells exhibited increases in fraction of cells in the G1 phase and decreases in fraction in S phase. Cell cycle distributions were determined by incorporation of BrdU for 20 min, anti-BrdU and propidium iodide staining, followed by flow cytometric analysis. Results of three independent experiments were averaged and standard deviations shown. **(B)** RNF4-depleted U2OS cells exhibited similar levels of CHK1 phosphorylation (Ser317) with or without treatment with 2 mM HU for 16 h. Levels were also measured in cells blocked in HU for 16 h then released into normal medium for various times. **(C)** RNF4-depleted HCT116 cells exhibited similar cell cycle distributions compared to control-depleted cells with or without treatment with HU, caffeine, or both. Results of three independent experiments were averaged and standard deviations shown.

The lack of a correlation between DSBs and HU sensitivity left unanswered the question of how RNF4 protects cells from prolonged DNA replication stress. To further investigate the roles of RNF4 in DNA synthesis under normal and replication-stress conditions, we examined the cell cycle profiles of control and RNF4-depleted HeLa, U2OS, and HCT116 cells by flow cytometry. For these experiments, we included the colon cancer cell line HCT116, because it has defects in the expression of the MRE11-RAD50-NBS1 complex and is thus more sensitive to replication stress. In the absence of DNA replication stress, RNF4-depleted cells exhibited reproducible perturbations of the G1 and S cell cycle phases in U2OS and HCT116 cells, showing a higher fraction of cells in G1 and a lower fraction in S phase compared to control-depleted cells (Figure 2A; Supplementary Table S1A). RNF4-depleted U2OS cells exhibited 17% more cells in G1 and 15% less in S phase, and for HCT116, it was 14% more in G1 and 22% less in S phase. For HeLa cells, it was 5% more cells in G1 and 4% less in S phase, but the results were not significant. These results show cell type-specific sensitivities to RNF4 depletion with HeLa cells being relatively resistant and U2OS and HCT116 cells being sensitive.

To test whether the excess accumulation of cells in G1 in the absence of HU was due to a checkpoint response, we measured the phosphorylation of CHK1 (Ser317) in untreated cells and found no evidence of more or less DNA damage checkpoint activation compared to control-depleted U2OS cells (Figure 2B). Consistent with a lack of DNA damage checkpoint activation, treating HCT116 cells with the general checkpoint inhibitor caffeine had negligible effects on cell cycle distributions (Figure 2C; Supplementary Table S1B). Thus, the effect of RNF4 depletion on the cell cycle in untreated cells is not caused by activation of ATR or ATM by a DNA damage signal.

**FIGURE 3:**
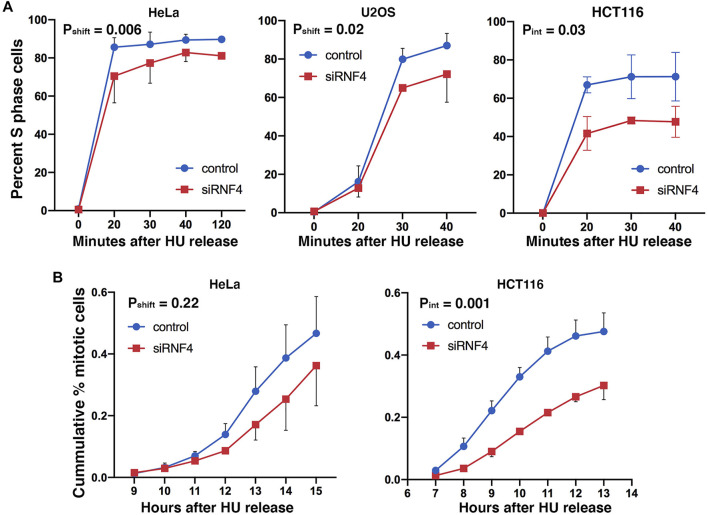
RNF4 depletion was associated with a delay in cell cycle progression after replication stress. **(A)** Graphs of percent of S phase cells determined by BrdU incorporation and flow cytometric analysis. RNF4- and control-depleted HeLa, U2OS, and HCT 116 cells were cultured in 2 mM HU for 16 h and subsequently released into normal medium and labeled with 20 μM BrdU for 20 min at different times after release from the HU block. Results of three independent experiments were averaged and standard deviations shown. Because error bar overlaps made the graphs difficult to view for some points, we have only shown the error bars in opposing directions. **(B)** Graphs of the cumulative percent of cells entering mitosis after release from HU block. RNF4- and control-depleted HeLa and HCT 116 cells were cultured in 2 mM HU for 16 h and then released into normal medium. Cells entering mitosis were scored by DAPI staining. Results of three independent experiments were averaged and standard deviations shown. Because error bar overlaps made the graphs difficult to view for some points, we have only shown the error bars in opposing directions.

We next investigated how RNF4 depletion affects the progression of cells through S phase by examining the kinetics of DNA synthesis resumption following release from prolonged HU treatment. We treated HeLa, U2OS, and HCT116 cells with 2 mM HU for 16 h and released them into normal medium containing BrdU. Cells were then analyzed by flow cytometry at different time points following HU release. This analysis revealed that RNF4-depleted cells treated with HU exhibited a delay in the incorporation of BrdU compared to control-depleted cells (Figure 3A), with HCT116 cells exhibiting the most severe defect. An effect of replication stress was also evidenced by a reduction in the total percent of RNF4-depleted cells that incorporated BrdU after HU release (6% reduction in HeLa cells, 13% reduction in U2OS cells, and 25% reduction in HCT116 cells at 40 min after HU release).

We also examined levels of CHK1 phosphorylation following release from overnight HU treatment. In both control- and RNF4-depleted cells, CHK1 phosphorylation remained elevated at least 150 min after release (Figure 2B), suggesting that dormant origin firing (see below) does not depend on reduction of CHK1 phosphorylation after HU release.

As an independent measure of the effect of RNF4 on S phase progression and exit, we treated HeLa and HCT116 cells with HU for 16 h, released the cells into normal medium, and then measured the mitotic index at different times after release. RNF4-depleted HeLa cells exhibited a delay in transit to metaphase following HU release compared to control-depleted cells, but the results were not significant (Figure 3B). HCT116 cells exhibited a more severe delay (>2 h), and a majority of cells appeared to fail to complete S phase. These results were consistent with the extreme hypersensitivity of HCT116 cells to RNF4 depletion (Supplementary Figure S2A). Thus, RNF4 is required for efficient resumption of DNA replication and completion of S phase following prolonged HU-induced DNA replication stress.

### RNF4 was Required for Activation of Dormant Origins After Replication Fork Collapse

**FIGURE 4:**
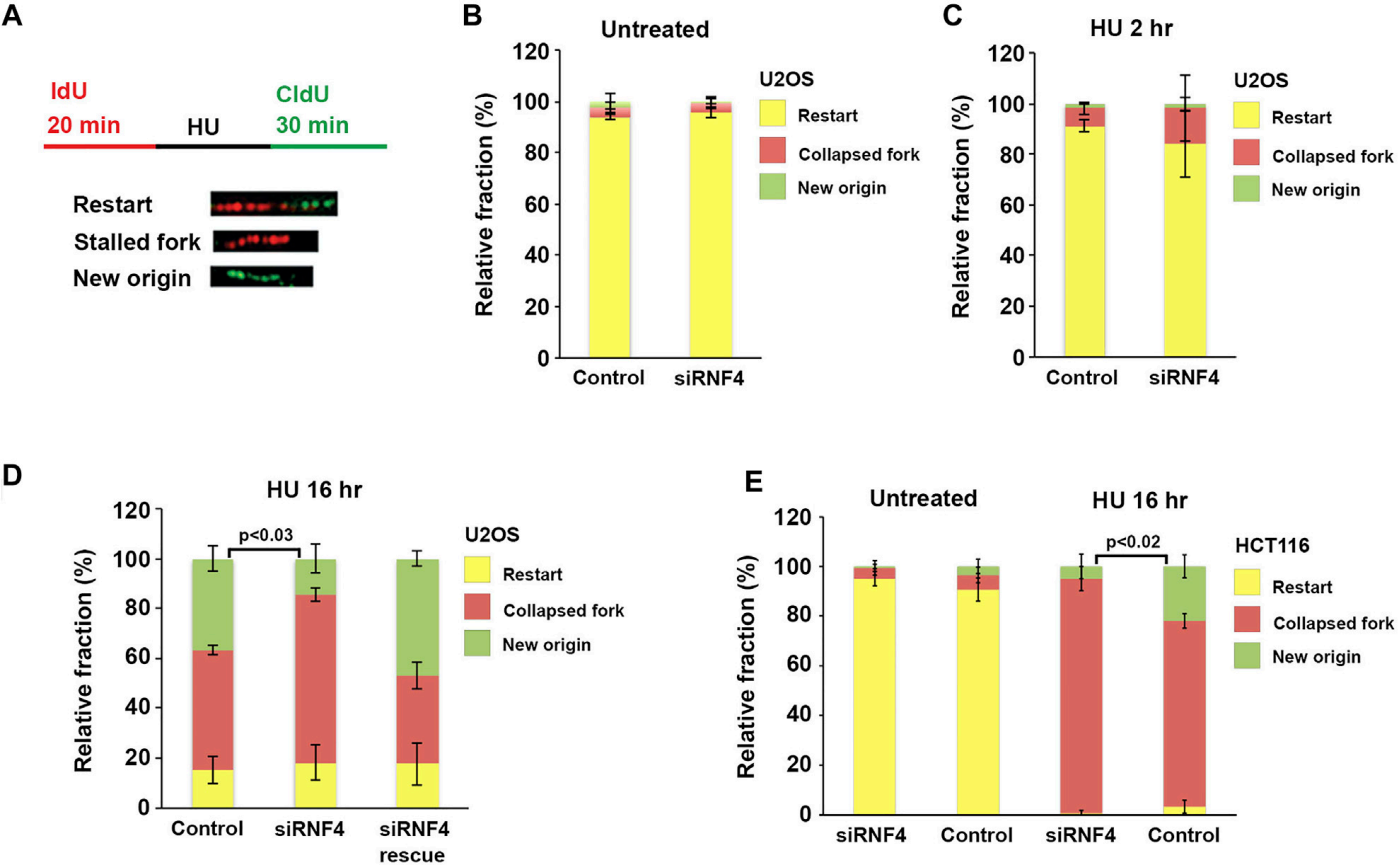
RNF4 depletion reduced dormant origin activation following prolonged replication stress. **(A)** Schematic representation of the dual labeling DNA fiber assay and possible outcomes. **(B)** Quantitative analysis of replication restart in control and RNF4-depleted U2OS cells in the absence of HU. Results from three independent experiments with standard deviations are shown. **(C)** Quantitative analysis of replication restart in control and RNF4-depleted U2OS cells following release from 2-h treatments with 2 mM HU. Results from three independent experiments with standard deviations are shown. **(D)** Quantitative analysis of replication restart after release from 16-h treatments with 2 mM HU. Cells transfected with RNF4-specific siRNAs together with an siRNA-resistant RNF4 cDNA showed complementation. Results from three independent experiments with standard deviations are shown.

The delay in resumption of DNA synthesis after prolonged HU treatment suggested that RNF4 may be required for the activation of dormant origins in proximity to collapsed replication forks. We therefore measured the effects of RNF4 depletion on replication dynamics using the DNA fiber assay. To carry out this assay, RNF4-depleted and control-depleted U2OS cells were incubated in medium supplemented with 20 μM IdU for 20 min. The IdU-containing medium was then replaced with medium containing 2 mM HU for 2 or 16 h, followed by release from HU and incubation in medium supplemented with 100 μM CldU for 30 min (Figure 4A). Cells were then processed for single molecule stretching and immunofluorescence detection of halogenated nucleotide incorporation. We then calculated the percentage of DNA molecules labeled with both IdU and CldU (representing replication fork restart), IdU only (representing irreversible fork collapse and termination of replication), or CldU only (representing replication from newly fired origins). In the absence of HU, >95% of labeled DNA molecules in both RNF4-depleted and control-depleted cells contained IdU and CldU labels (Figure 4B), indicating that RNF4 is not required for ongoing replication in the absence of replication stress.

In cells treated with HU for 2 hours, the increase in collapsed replication forks in RNF4-depleted cells compared to control-depleted cells was not significant (Figure 4C). Following treatment with HU for 16 h, RNF4-depleted and control-depleted cells also exhibited minimal differences in the percentage of replication forks undergoing restart. In contrast, however, RNF4-depleted cells exhibited a significantly higher percentage of collapsed forks and a lower percentage of forks starting at new origins compared to control-depleted cells (Figure 4D). This observed defect in new origin firing was rescued by the ectopic expression of siRNA-resistant *RNF4* mRNA. These results provided further evidence that RNF4 is required for efficient resumption of DNA synthesis following replication stress and pointed to a role in the activation of dormant origins following fork collapse.

### BLM was Found to be Regulated by RNF4 at Sites of Replication Stress

BLM accumulates at stalled and collapsed forks, is sumoylated in response to replication stress (Ouyang et al., 2009; Xiao et al., 2015), and it has been reported to be a substrate of RNF4 in proteomic studies (Kumar et al., 2017). We therefore hypothesized that RNF4 may regulate ubiquitin-mediated turnover of BLM at collapsed forks, and that an accumulation of excess BLM in the absence of RNF4 may inhibit the normal resumption of DNA synthesis after prolonged HU treatment.

**FIGURE 5:**
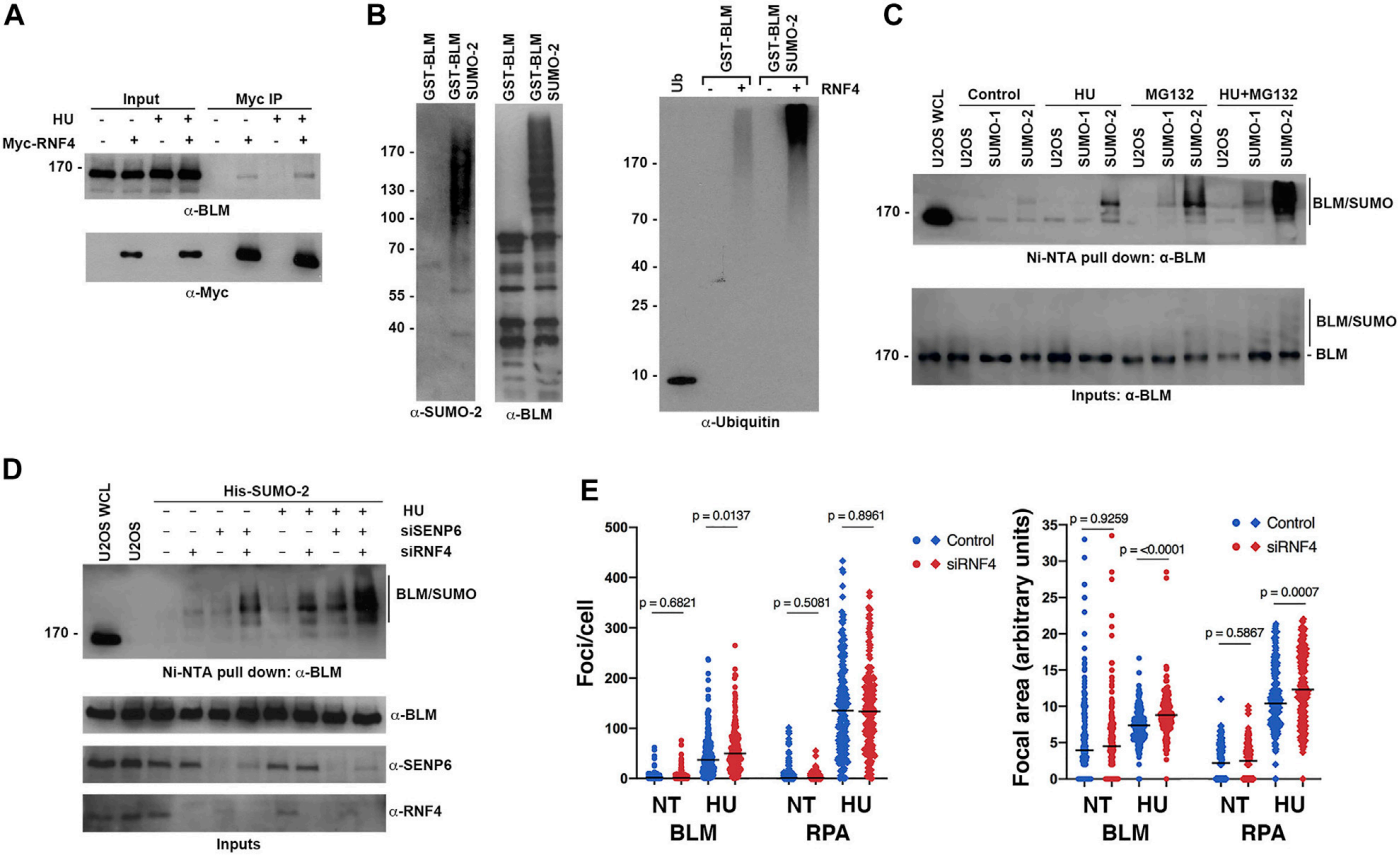
RNF4 modulated BLM levels at sites of replication stress. **(A)** HeLa cells were transfected with Myc-tagged RNF4 and cultured in the presence or absence of HU. RNF4 was immunopurified from cell lysates and protein complexes were analyzed by immunoblot analysis with anti-Myc and anti-BLM antibodies. **(B)** A purified GST-tagged N-terminal fragment of BLM (amino acids 1- 431) modified by SUMO-2 *in vitro* is ubiquitylated by RNF4 *in vitro* whereas unmodified BLM is not (right panel). Ubiquitin alone (Ub). Input levels of GST-BLM and GST-BLM-SUMO2 were determined by immunoblot analysis (two left panels). **(C)** Levels of SUMO-BLM increase with 2 mM HU treatment for 12 h, treatment with MG132 for 3 hours, or treatment with HU for 12 h followed by MG132 for 3 h. Pull-downs with Ni-NTA agarose beads were performed in U2OS cells that express a His-tagged SUMO1 or SUMO2. U2OS WCL = control U2OS whole cell lysate. **(D)** Levels of SUMO-BLM increase in U2OS cells depleted of RNF4, SENP6, or both, in response to 2 mM HU for 12 h or in the absence of treatment. Ni-NTA pull-downs shown as in **(C)**. **(E)** Excess BLM and RPA proteins accumulated at collapsed forks in RNF4-depleted HeLa cells treated with 2 mM HU for 16 h compared to control-depleted cells. Left panel, enumerations of BLM and RPA foci. Right panel, quantifications of focal areas of BLM and RPA foci. Results from three independent experiments were combined. NT, not treated.

To begin to test this hypothesis, we first investigated whether RNF4 interacts with BLM and whether this interaction is regulated by HU-induced replication stress. HeLa cells were transfected with a Myc-RNF4 expression construct and Myc-RNF4 was immunopurified from cell lysates prepared from control and HU-treated cells. Immunoblot analysis with BLM antibodies revealed an interaction in the absence of HU that increased following HU treatment (Figure 5A). The predominant form of BLM detected in the pulldown was unsumoylated BLM, suggesting a possible direct interaction between BLM and RNF4. Next, we tested whether sumoylated BLM is ubiquitylated by RNF4 by performing *in vitro* conjugation assays using purified recombinant proteins. Using an N-terminal fragment of GST-tagged BLM (BLM 1-431) that is readily sumoylated *in vitro* (Zhu et al., 2008), we found that sumoylated BLM was robustly ubiquitylated in comparison to unmodified BLM (Figure 5B).

To further investigate whether sumoylation targets BLM for ubiquitin-mediated turnover *in vivo*, we analyzed levels of sumoylated BLM in U2OS cell lines that stably expressed either a His-tagged SUMO-1 or His-tagged SUMO-2 using nickel-NTA bead affinity pull down and immunoblot analysis. As anticipated from previous studies (Eladad et al., 2005; Zhu et al., 2008; Ouyang et al., 2009), BLM was preferentially modified by SUMO-2 at low levels under control conditions, and these levels increased in response to HU treatment (Figure 5C). Consistent with sumoylation functioning as a signal for proteasome-mediated turnover, levels of sumoylated BLM were greater in cells treated with MG132 compared to untreated cells, and sumoylated BLM levels were further increased in cells treated with both HU and MG132 (Figure 5C). We note that the ratio of sumoylated to unsumoylated BLM was low, even under conditions of HU and MG132 treatment (Figure 5C).

To test whether RNF4 regulates the turnover of sumoylated BLM, we next measured sumoylated BLM levels in RNF4-depleted cells. We also tested cells depleted for SENP6, a chain-editing SUMO isopeptidase capable of limiting poly-sumoylation and thereby RNF4 recognition (Mukhopadhyay et al., 2010). In untreated cells, RNF4 depletion had minimal effect on sumoylated BLM levels, whereas levels were significantly increased when RNF4 was depleted in HU-treated cells (Figure 5D). Similarly, SENP6 depletion alone had minimal effect on sumoylated BLM levels in untreated cells, whereas levels were increased in combination with HU treatment. In comparison to the single knockdowns, an increase in sumoylated BLM levels was observed in cells co-depleted of both SENP6 and RNF4, and the highest levels of sumoylated BLM were detected in co-depleted cells treated with HU (Figure 5D).

Direct investigation of kinetics of sumoylated BLM turnover in control and RNF4-depleted cells was complicated by the low ratio of modified to unmodified BLM. In addition, studies using inhibition of new protein synthesis with cycloheximide were not possible because RNF4 is itself turned over within 2 h of cycloheximide addition (Supplementary Figure S3A).

Finally, to investigate the effect of RNF4 on BLM accumulation at sites of collapsed DNA replication forks, we quantified the number and area of BLM foci in control and RNF4-depleted cells by indirect immunofluorescence confocal microscopy (Figure 5E; Supplementary Figure S3B). In the absence of HU, we found that the number and area of BLM foci were similar in RNF4- and control-depleted cells. In contrast, following HU treatment for 16 h, both the number of BLM foci and their focal areas were significantly greater in RNF4-depleted cells compared to control-depleted cells. Because RPA is also sumoylated and accumulates at collapsed replication forks (Dou et al., 2010), we quantified RPA foci and focal areas in both untreated and treated cells. Although the numbers of RPA foci remained the same, the areas of RPA foci were significantly greater in RNF4-depleted cells compared to control-depleted cells following HU treatment. Altogether, these findings demonstrated that sumoylated BLM is a substrate for RNF4-mediated ubiquitylation and proteasome-mediated turnover, and that BLM accumulates at sites of collapsed DNA replication forks in the absence of RNF4.

### BLM Contributed to Suppression of Dormant Origin Firing in RNF4-Depleted Cells

**FIGURE 6:**
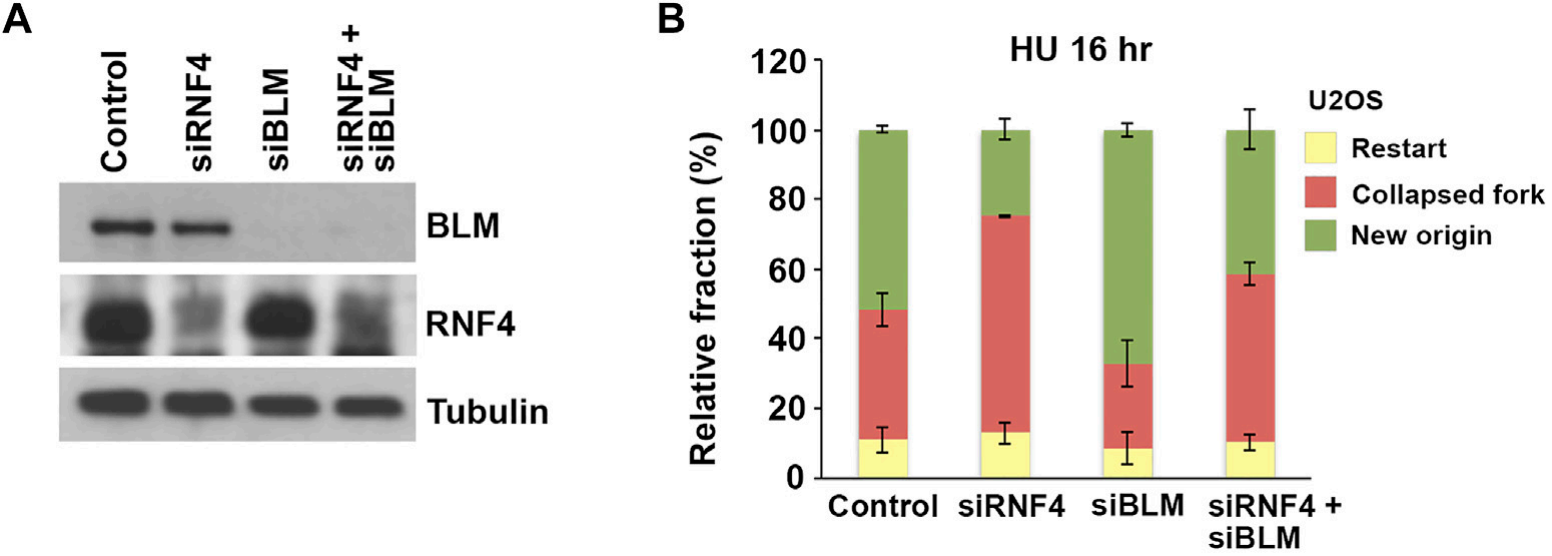
BLM depletion rescued effects of RNF4 depletion on DNA replication stress responses. **(A)** Immunoblot analysis of U2OS cell lysates following siRNA-mediated depletion of RNF4, BLM, or BLM together with RNF4. **(B)** Quantitative analysis of replication restart in U2OS control, RNF4-depleted, BLM-depleted, or RNF4 and BLM co-depleted cells after release from 16-h 2 mM HU treatments. Results from three independent experiments with standard deviations are shown.

To investigate whether the accumulation of BLM at collapsed replication forks in RNF4-depleted cells contributed to defects in replication restart, we next asked whether co-depletion of BLM could rescue the observed defects in dormant origin firing. We again used DNA fiber assays to analyze replication dynamics in U2OS cells co-depleted of BLM and RNF4 (Figure 6A). Consistent with previous findings (Davies et al., 2007), we observed an increase in the proportion of DNA replication restart events from new origins in BLM-depleted cells after release from treatment with HU for 16 h compared to control-depleted cells (Figure 6B). In addition, we again observed a decrease in replication restart from new origins in RNF4-depleted cells. In contrast, replication restart from new origins in cells co-depleted of RNF4 and BLM was similar to control-depleted cells, which demonstrated that BLM depletion rescued the defect in dormant origin firing observed in RNF4-depleted cells. These findings were consistent with our hypothesis that an accumulation of excess BLM at collapsed replication forks inhibits the normal resumption of DNA replication following prolonged HU treatment.

## Discussion

The experiments presented here have implicated RNF4 function in recovery from fork collapse. Brief exposure of RNF4-depleted cells to HU elicited little effect on replication fork stability, whereas RNF4-depleted cells exposed to prolonged HU treatment—a treatment that causes widespread fork collapse—exhibited a delay in the resumption of DNA synthesis after removal of drug and an increase in the percentage of cells that permanently left the cell cycle, which was demonstrated in both flow cytometric and DNA fiber assays (Figures 3, 4). Extending the results of earlier proteomic studies (Kumar et al., 2017), we showed that BLM interacts with RNF4, sumoylated BLM is ubiquitylated by RNF4 *in vitro*, and RNF4 depletion led to a substantial increase in SUMO-BLM in HU treated cells (Figure 5). Moreover, depletion of RNF4 led to excessive accumulation of BLM and RPA at collapsed forks as evidenced by increased focal areas. The observation that the number of RPA foci per cell remained the same whilst the number of BLM foci per cells increased could be an effect of better detection, as increased BLM protein accumulation at collapsed forks may have brought more sites of collapsed forks at which BLM localized above the detection threshold. The generation of excess SUMO-BLM in RNF4-depleted, HU-treated cells could explain the observed increases in BLM focal areas due to higher-order interactions, because BLM contains both multiple SUMO acceptors sites and a SIM and BLM can form a multimer *in vitro* (Karow et al., 1999; Xu et al., 2012). Earlier studies showed that the SUMO E2 conjugating enzyme UBC9 and E3 ligase NSMCE2 are targets for RNF4 that enforce a negative regulatory loop (Kumar et al., 2017). *In vitro*, UBC9 is sufficient for poly-sumoylation or multi-mono-sumoylation of BLM (Eladad et al., 2005) and we showed recently that NSMCE2 is required for sumoylation of BLM and for accumulation of BLM at collapsed replication forks in response to HU treatment (Pond et al., 2019). Although we cannot rule out the possibility that BLM accumulates excessively in HU-treated, RNF4-depleted cells due to damage at the replication fork, we favor the hypothesis that the excessive accumulation of BLM has pathological effects on recovery of DNA synthesis after prolonged HU exposure and that, by controlling the levels of sumoylated BLM, RNF4 facilitates resumption of DNA synthesis after widespread fork collapse. We previously showed that SD-BLM accumulates excessively at stressed replication forks, suggesting this accumulation is an upstream event. We found here that the delay in resumption of DNA synthesis in RNF4-depleted cells was rescued by co-depletion of the BLM protein (Figure 6).

The mechanism by which RNF4 contributes to immediate resumption of DNA synthesis is not known. One possibility is that the excess SUMO-BLM that accumulates at collapsed forks in RNF4-depleted cells ties up large amounts of the unsumoylated BLM and that BLM needs to be released and recycled in order to achieve efficient dormant origin firing or resolution of fork impediments that prevent rapid replisome take off at already-fired dormant origins. We do note, however, that the ratio of sumoylated to unsumoylated BLM is low even under conditions that limit its turnover (RNF4 and SENP6 knockdown and MG132 treatment). These low levels are nonetheless consistent with functionally relevant levels of sumoylation observed with other SUMO substrates (Hay, 2005). Another possibility is that RNF4-mediated degradation of SUMO-BLM at collapsed forks drives the disassembly of multiple factors in repair foci that are needed for dormant origin firing or replisome take off. Moreover, we do not know that BLM is the only sumoylated protein whose depletion would result in rescue. Because multiple damage-response factors are sumoylated, it is possible that depletion of other sumoylated proteins at sites of replication stress could also rescue the DNA synthesis resumption defect. In particular, interesting candidates would be proteins that contain both SUMO acceptor sites and SIMs, such as SLX4, which could mediate higher-order interactions and focus formation (Bursomanno et al., 2015; Hendriks et al., 2015; Gonzalez-Prieto et al., 2021).

Short-term treatment of *BLM*-deficient cells with HU induces excessive fork collapse and dormant-origin firing relative to normal cells (Davies et al., 2007; Patel et al., 2017). This evidence argues that BLM itself does not play a direct role in activating dormant origin firing, nor has it a known activity in the firing of origins of replication in early or late periods of S phase from *in vitro* studies (On et al., 2014; Kurat et al., 2017). For these reasons, we do not favor a hypothesis that places SUMO-BLM in an inhibitory role in firing of dormant origins.

Because RNF4 depletion promotes an increase in the size and numbers of PML bodies (Lallemand-Breitenbach et al., 2008; Tatham et al., 2008), it was formally possible that the flux of trafficking of damage response proteins that normally accumulate in PML bodies due to sumoylation or SIMs could be delayed by RNF4 depletion. In untreated RNF4-depleted cells, there is an increase in BLM foci approximately corresponding to the increase in PML bodies (Bohm et al., 2015). BLM’s localization to PML bodies relies primarily on its SIM (Eladad et al., 2005; Zhu et al., 2008); however, we did not find evidence that BLM recruitment to sites of replication stress was less efficient, because BLM substantially co-localized with RPA foci after HU treatment and RPA is not a PML-associated nuclear protein.

RNF4 is important in the repair of DSBs, because RNF4-depleted cells are hypersensitive to *γ*-radiation and have a defect in the recruitment of RAD51 to DSBs (Galanty et al., 2012; Yin et al., 2012; Vyas et al., 2013). Our investigation began with the question concerning the hypersensitivity of RNF4-depleted cells to HU treatment and with the hypothesis that RNF4 would be important for the repair of replication-associated DSBs (Figure 1). However, we did not observe a role for RNF4 in recruitment of RAD51 to collapsed replication forks caused by prolonged HU treatment. RNF4 depletion did not impair ATR- or ATM-dependent checkpoint signaling in response to HU, as indicated by normal *γ*-H2AX levels, nor were levels of phosphorylated CHK1 affected by RNF4 depletion with or without HU treatment (Figure 2B). Levels of SCE were similar in RNF4-depleted compared to control-depleted cells with or without HU treatment (Figure 1F). To our surprise, RNF4-depleted cells were not hypersensitive to CPT, and levels of CPT-induced DSBs were unaffected by RNF4 depletion (Figures 1C,D), despite the role of RNF4 in degradation of topoisomerase I-DNA cleavage complexes (Sun et al., 2020). Treatment with CPT generates a predominance of single-ended, replication-associated DSBs, and our evidence indicates that the repair of these breaks is not affected by RNF4 depletion. Because excess BLM accumulated at collapsed forks without affecting the rate of SCEs, our results also excluded a hypothesis in which BLM or other RNF4-regulated HR factors must be extracted from collapsed forks in order for HR repair to proceed. These data highlight the importance of damage context. Previous evidence has shown that the requirements for recruitment of RAD51 to two-ended DSBs and stalled forks are different (Chaudhuri et al., 2016). Moreover, previous results have shown that cells held in HU for up to 24 h do not accumulate many DSBs (Petermann et al., 2010); instead, DSBs accumulate after release from HU blockade (Pond et al., 2019) or after longer treatments with HU. Some fraction of the DSBs that accumulate after release from HU occur in late S phase, indicating that breaks occur when active forks converge on collapsed forks. The SCE results shown here indicate that repair of these DSBs was normal in RNF4-depleted cells. Our results did not address the question whether RNF4 was required for the repair of breaks generated during mitosis or at cytokinesis, where two-ended DSBs are thought to be generated (Spies et al., 2019).

In mouse knockout studies, RNF4 was found to be essential for embryogenesis and *Rnf4* ^*-/-*^ mouse embryonic fibroblast lines could not be obtained in at least one study (Hu et al., 2010; Vyas et al., 2013). *RNF4* ^*-/-*^ chicken DT40 cells are viable, but they have limited proliferation capacity due to chromosomal loss (Yin et al., 2012; Hirota et al., 2014); *RNF4* knock-out human cell lines generated using CRISPR technology have been reported (Maure et al., 2016; Sun et al., 2020), which suggests that RNF4 is not cell essential. However, colony survival assays have consistently shown a ∼50% reduction in RNF4-depleted cells relative to control, indicating that RNF4 has a role in cell viability. Similarly, BLM-deficient cells proliferate less robustly than normal cells. These observations are consistent with the synthetic lethality of the yeast orthologs of *BLM* and *RNF4*, namely, *SGS1* and the *SLX5*-*SLX8* complex (Mullen et al., 2001). We found that RNF4-depleted cells exhibited an increase in the fraction of cells in the G1 phase and showed evidence that this increase was not a result of a DNA damage signal (Figure 2). Although it has been proposed that the essential function of RNF4 is due to its role in maintaining genomic integrity, RNF4 also plays important roles in gene transcription (Moilanen et al., 1998), global DNA methylation levels (Hu et al., 2010), chromatin structure (Hendriks et al., 2015), and regulation of oncogenes (Thomas et al., 2016), and it could be the combination of all these roles that leads to loss of viability in untreated cells.

The present study showed that the process of replication fork collapse and dormant origin firing are connected through the action of RNF4. RNF4 is required for the clearance of BLM from collapsed forks and the failure to release BLM from collapsed forks affected the recovery of cells from prolonged replication stress. With the varied roles that RNF4 plays in DNA damage responses, further investigation into its efficacy as a potential target in cancer treatments seems warranted. As a cancer target, the function of RNF4 in turnover of sumoylated BLM and other HR proteins could perhaps be utilized to slow recovery in replication stressed cells.

## Data Availability

The raw data supporting the conclusion of this article will be made available by the authors, without undue reservation.

## Supplementary Material

Supplementary Figure S1
**(A)** Bar graphs showing results of clonogenic survival assays on RNF4-depleted and control-depleted U2OS cells exposed to varying concentrations of HU. Results from three independent assays were averaged and standard deviations shown. **(B)** Bar graphs showing results of clonogenic survival assays on RNF4-depleted and control-depleted U2OS cells exposed to varying concentrations of camptothecin (CPT) for three hours. Results from three independent assays were averaged and standard deviations shown. **(C)** Bar graphs showing the relative levels of DSBs in RNF4-depleted and control-depleted HeLa cells exposed to varying concentrations of CPT for three hours, as determined by pulsed field gel electrophoresis. Induced DSBs were normalized to DSBs in untransfected and untreated cells. Results from three independent experiments were averaged and standard deviations shown. **(D)** Box and whiskers plot showing the quantifications of focal areas of RAD51 focal accumulations, in RNF4-depleted and control-depleted cells with and without treatment with 2 mM HU for 16 h. RAD51 localized to PML nuclear bodies in untreated cells, and PML bodies have been noted to enlarge in RNF4-depleted cells. Results from three independent experiments were combined. NT, not treated.

Supplementary Figure S2 Proliferation defect in RNF4-depleted HCT116 cells exposed to 2 mM HU for 16 h. Results from three independent experiments with standard deviations are shown.

Supplementary Figure S3
**(A)** BLM stability was unaffected by RNF4 depletion in HeLa cells treated with cycloheximide. Cycloheximide was added at time zero and treated cells examined hourly for six hours. Untreated control examined at six hours is indicated by an asterisk. Note that RNF4 levels were reduced by more than half after 2 h. **(B)** Representative indirect immunofluorescence image of BLM and RPA foci detected in HeLa cells transfected with negative control and RNF4 siRNAs and treated with 2 mM HU for 16 h.
