# Supplementary figures and images for: RNF4 Regulates the BLM Helicase in Recovery From Replication Fork Collapse
Source: Front Genet. 2021 Nov 12;12:753535. doi: 10.3389/fgene.2021.753535 (PMC8633118; doi:10.3389/fgene.2021.753535)

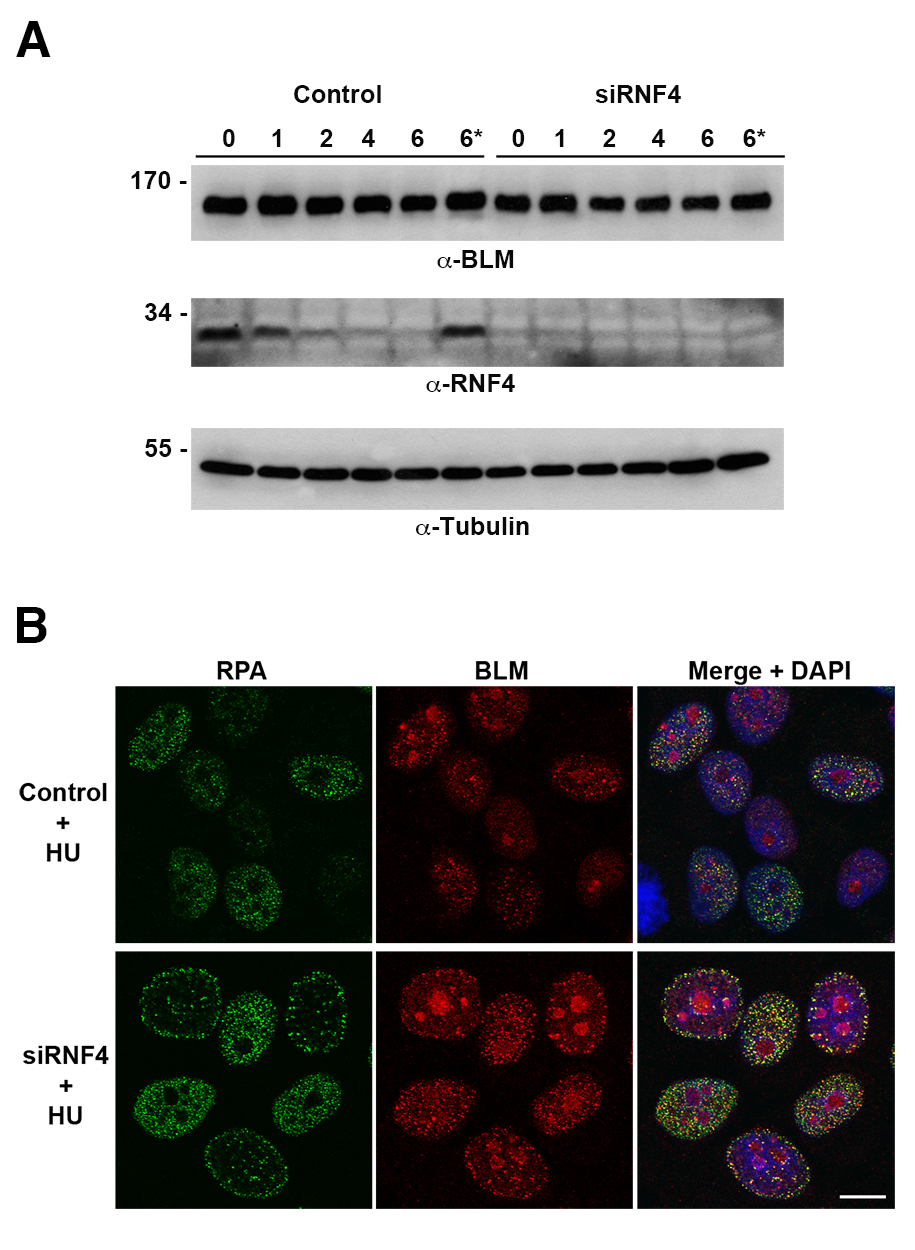

Supplement: Supplementary file 1 [file Image3.JPEG]

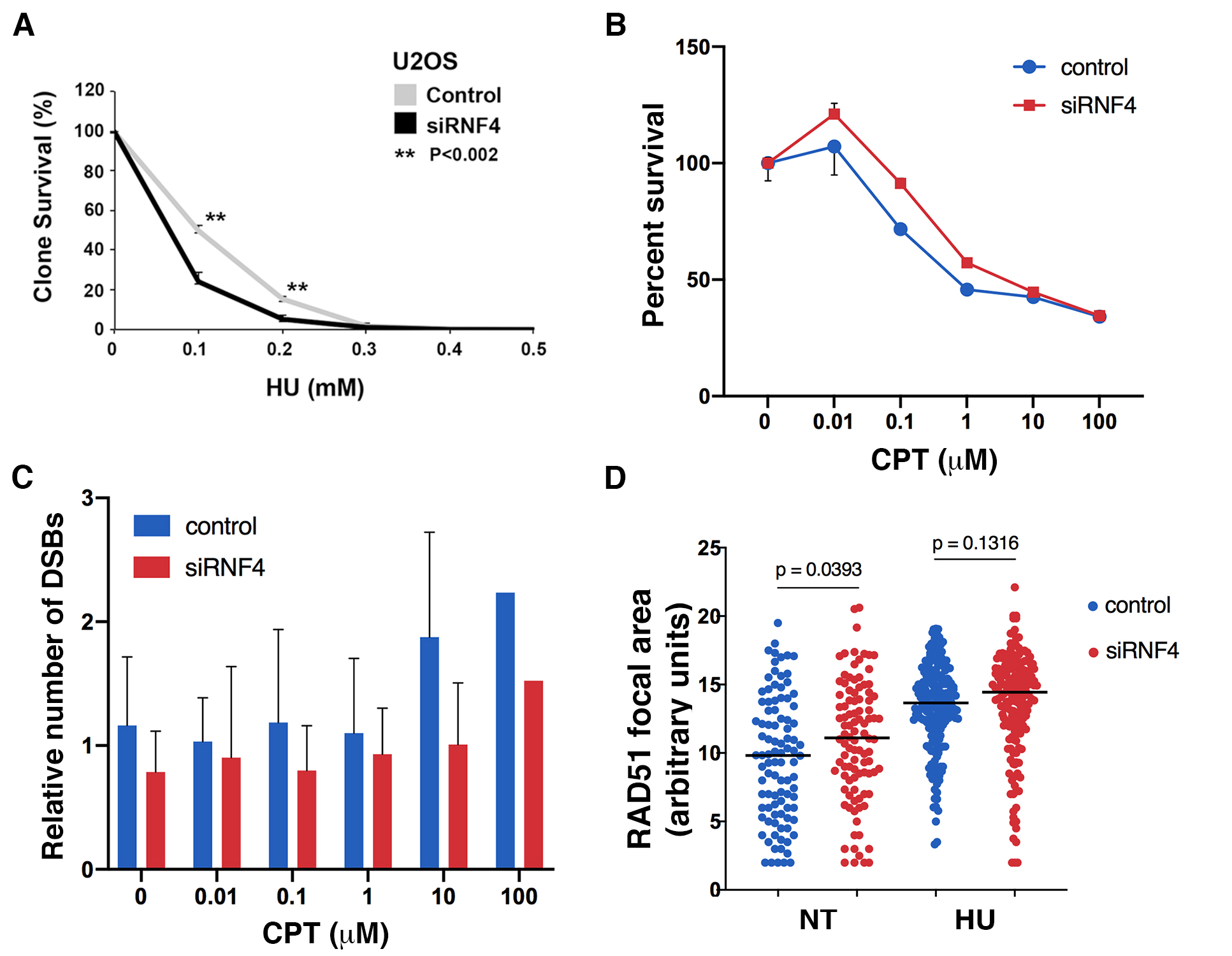

Supplement: Supplementary file 3 [file Image1.JPEG]

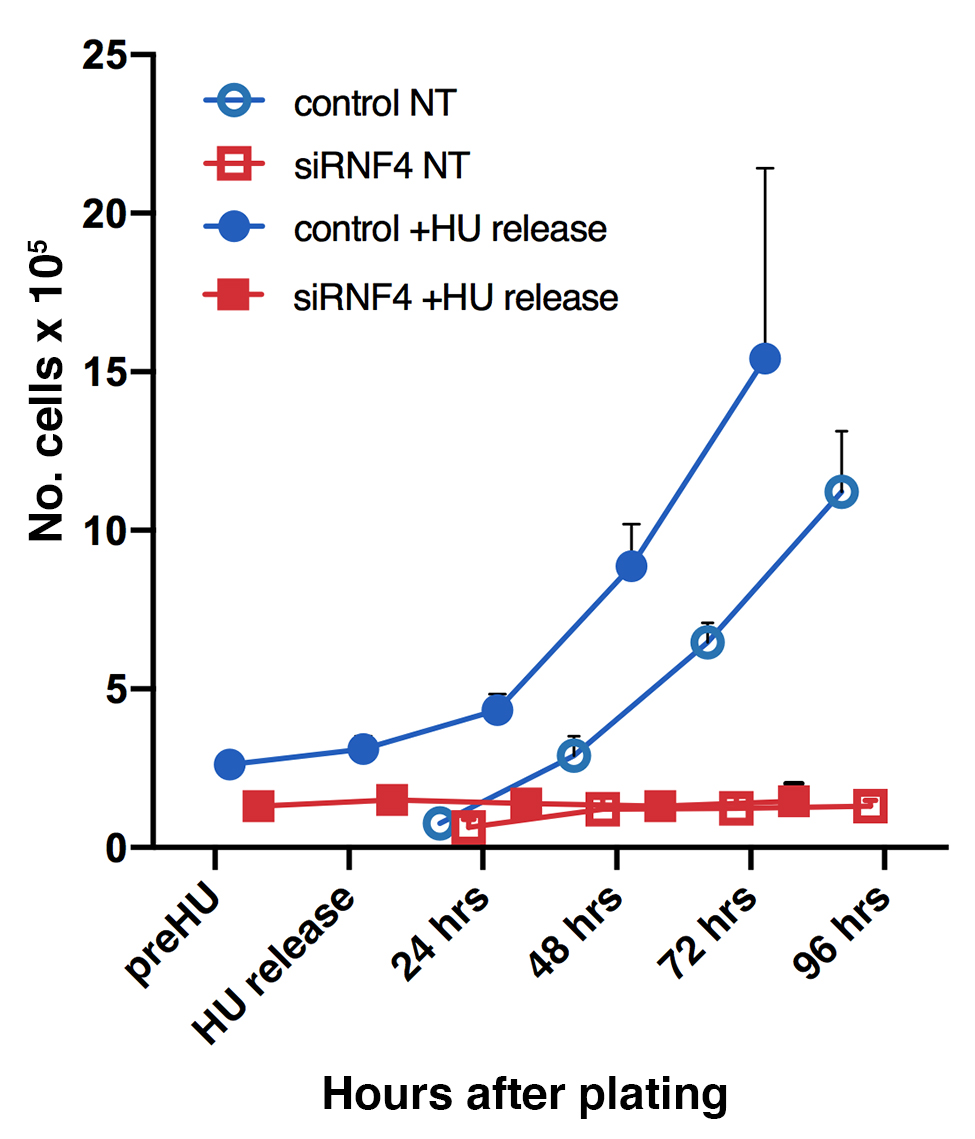

Supplement: Supplementary file 4 [file Image2.JPEG]
