# Supplementary material for: RNF4 Regulates the BLM Helicase in Recovery From Replication Fork Collapse
Source: Front Genet. 2021 Nov 12;12:753535. doi: 10.3389/fgene.2021.753535 (PMC8633118; doi:10.3389/fgene.2021.753535)
Supplement: Supplementary file 2 [file Table1.DOCX]

Supplementary Table 1A. Statistical significance of cell cycle profile differences between RNF4 and control depletions in the absence of hydroxyurea treatment.

| **Cell line** |  | **Mean (S.E.)** | | **p-value for the difference** |
| --- | --- | --- | --- | --- |
|  |  | **NC1** | **RNF4** |  |
| HeLa | % G1 | 56.36 (1.84) | 61.26 (1.85) | 0.13 |
|  | % S | 30.99 (1.68)) | 26.63 (1.67) | 0.14 |
|  | % G2 | 12.16 (0.79) | 11.59 (0.79) | 0.63 |
|  |  |  |  |  |
| U2OS | % G1 | 45.15 (2.21) | 62.13 (2.22) | 0.0016 |
|  | % S | 45.43 (2.19) | 28.40 (2.18) | 0.0015 |
|  | % G2 | 8.07 (0.86) | 7.93 (0.87) | 0.91 |
|  |  |  |  |  |
| HCT116 | % G1 | 41.77 (4.11) | 55.83 (4.12) | 0.07 |
|  | % S | 37.63 (1.93) | 15.86 (1.93) | 0.001 |
|  | % G2 | 16.87 (3.84) | 23.27 (3.85) | 0.30 |
|  |  |  |  |  |

Supplementary Table 1B. Statistical significance of cell cycle profile differences between caffeine and hydroxyurea treatments in RNF4 and control depletions.

|  |  | **p-value for interaction** | **p-value for the** |
| --- | --- | --- | --- |
|  | **Condition** | **caffeine*RNF4** | **difference between caffeine treatment** |
| % G1 | no HU | 0.98 | 0.07 |
|  | HU | 0.51 | 0.82 |
|  |  |  |  |
| % S | no HU | 0.94 | 0.37 |
|  | HU | 0.83 | 0.04 |
|  |  |  |  |
| % G2 | no HU | 0.98 | 0.77 |
|  | HU | 0.97 | 0.03 |
